# Nomogram for prediction of severe community-acquired pneumonia development in diabetic patients: a multicenter study

**DOI:** 10.1186/s12890-022-02183-9

**Published:** 2022-11-07

**Authors:** Ruoming Tan, Bing Liu, Chunliu Zhao, Junhai Yan, Tingting Pan, Min Zhou, Hongping Qu

**Affiliations:** 1 Department of Critical Care Medicine, Ruijin Hospital, Shanghai Jiaotong University School of Medicine, Shanghai, China; 2 Department of Pulmonary and Critical Care Medicine, Ruijin Hospital, Shanghai Jiao Tong University School of Medicine, Shanghai, China; 3 Shanghai Key Laboratory of Emergency Prevention, Diagnosis, and Treatment of Respiratory Infectious Diseases, Shanghai, China; 4 Institute of Respiratory Diseases, Shanghai Jiaotong University School of Medicine, Shanghai, China; 5 Department of Respiratory Medicine, Ruijin Hospital Luwan Branch, Shanghai Jiao Tong University School of Medicine, Shanghai, China

**Keywords:** Prediction, Severe community-acquired pneumonia, Diabetes mellitus, Nomogram

## Abstract

**Background:**

Diabetic patients with community-acquired pneumonia (CAP) have an increased risk of progressing to severe CAP. It is essential to develop predictive tools at the onset of the disease for early identification and intervention. This study aimed to develop and validate a clinical feature-based nomogram to identify diabetic patients with CAP at risk of developing severe CAP.

**Method:**

A retrospective cohort study was conducted between January 2019 to December 2020. 1026 patients with CAP admitted in 48 hospitals in Shanghai were enrolled. All included patients were randomly divided into the training and validation samples with a ratio of 7:3. The nomogram for the prediction of severe CAP development was established based on the results of the multivariate logistic regression analysis and other predictors with clinical relevance. The nomogram was then assessed using receiver operating characteristic curves (ROC), calibration curve, and decision curve analysis (DCA).

**Results:**

Multivariate analysis showed that chronic kidney dysfunction, malignant tumor, abnormal neutrophil count, abnormal lymphocyte count, decreased serum albumin level, and increased HbA1c level at admission was independently associated with progression to severe CAP in diabetic patients. A nomogram was established based on these above risk factors and other predictors with clinical relevance. The area under the curve (AUC) of the nomogram was 0.87 (95% CI 0.83–0.90) in the training set and 0.84 (95% CI 0.78–0.90). The calibration curve showed excellent agreement between the predicted possibility by the nomogram and the actual observation. The decision curve analysis indicated that the nomogram was applicable with a wide range of threshold probabilities due to the net benefit.

**Conclusion:**

Our nomogram can be applied to estimate early the probabilities of severe CAP development in diabetic patients with CAP, which has good prediction accuracy and discrimination abilities. Since included biomarkers are common, our findings may be performed well in clinical practice and improve the early management of diabetic patients with CAP.

## Introduction

Diabetes mellitus is a common chronic disease with multiple complications and contributes to the global health care burden. It is estimated that in 2011, 366 million patients with diabetes were reported worldwide, and by 2030, the number of patients with diabetes will increase by 50% [1]. In addition, diabetes is reported as one of the ten leading causes of death in the United States.

Infection is one of the severe complications in patients with diabetes, and the incidence varies between 32.7% and 90.5% [2, 3]. The lower respiratory tract is considered the most common site involved with infection in diabetic patients [4, 5]. Due to the immunocompromised state, patients with diabetes appear more susceptible to pneumonia than patients without diabetes. Besides, these patients have an increased risk of hyperglycemia, impaired lung function, and other chronic complications such as heart disease, renal failure, and pulmonary microangiopathy [6–8]. Recent studies demonstrated that diabetes is an independent risk factor for developing severe community-acquired pneumonia (CAP) and is associated with pneumonia-related hospitalization and mortality [7, 9–11]. Hence, based on those mentioned above, developing a predictive tool for progressing CAP to severe CAP in patients with diabetes may help improve the management of diabetic patients with CAP.

Unfortunately, studies have shown that diabetic patients have reduced predictive performance for CURB-65 and PSI grades compared to non-diabetic patients [12, 13]. Future studies should develop predictive models for assessing the severity of CAP in diabetic populations. This study aimed to identify potential predictors for the development of severe CAP in diabetes mellitus (DM) patients and to derive and validate a nomogram model to predict timely and appropriate treatment.

## Methods

### Design and setting

A retrospective cohort study was conducted between January 2019 to December 2020. 1026 patients with CAP admitted in 48 hospitals in Shanghai were enrolled. Institutional review board approval was obtained in accordance with local requirements. Informed consent from study patients was not required because of the retrospective design of the cohort study.

### Patient selections and definitions

All included patients were randomly divided into the training and validation samples with a ratio of 7:3. The basic characteristics of the two cohorts are listed in Table 1. CAP was defined by the following criteria: (1) acute lower respiratory tract infection with at least two symptoms (e.g., fever, cough or sputum production, dyspnea, chest pain); (2) new focal signs on physical examination of the chest; (3) new infiltrates on chest x-ray [14].

**Table 1:** Baseline characteristics of the patients in the training set and testing set

Characteristic	level	Testing set (n = 308)	Training set (n = 718)	p
**Demographic data**				
Sex [n(%)]	Female	132 (42.9)	311 (43.3)	0.947
	Male	176 (57.1)	407 (56.7)	
Age [n(%)]	< 65	101 (32.8)	260 (36.2)	0.327
	≥ 65	207 (67.2)	458 (63.8)	
Current smoker [n(%)]	0	228 (74.3)	536 (74.8)	0.931
	1	79 (25.7)	181 (25.2)	
Alcohol abuse [n(%)]	0	285 (92.8)	667 (93.0)	1.000
	1	22 ( 7.2)	50 ( 7.0)	
Temperature < 36.3 °C or ≥ 37.2 °C [n(%)]	0	45 (14.6)	115 (16.0)	0.635
	1	263 (85.4)	603 (84.0)	
**Comorbid condition**				
Hypertension [n(%)]	0	143 (46.4)	323 (45.0)	0.721
	1	165 (53.6)	395 (55.0)	
Chronic respiratory disease [n(%)]	0	246 (79.9)	600 (83.6)	0.181
	1	62 (20.1)	118 (16.4)	
Cardiac dysfunction [n(%)]	0	248 (80.5)	588 (81.9)	0.666
	1	60 (19.5)	130 (18.1)	
Cerebrovascular disease [n(%)]	0	262 (85.1)	613 (85.4)	0.974
	1	46 (14.9)	105 (14.6)	
Chronic kidney dysfunction [n(%)]	0	278 (90.3)	665 (92.6)	0.252
	1	30 ( 9.7)	53 ( 7.4)	
Rheumatic disorders [n(%)]	0	301 (97.7)	703 (97.9)	1.000
	1	7 ( 2.3)	15 ( 2.1)	
Malignant tumor [n(%)]	0	289 (93.8)	670 (93.3)	0.866
	1	19 ( 6.2)	48 ( 6.7)	
Long-term glucocorticoid medication [n(%)]	0	297 (96.4)	698 (97.2)	0.635
	1	11 ( 3.6)	20 ( 2.8)	
**Laboratory data**				
Neu < 2 or ≥ 7*10^9/L [n(%)]	0	161 (52.3)	386 (53.8)	0.712
	1	147 (47.7)	332 (46.2)	
L < 0.8 or ≥ 4*10^9/L [n(%)]	0	222 (72.1)	505 (70.3)	0.625
	1	86 (27.9)	213 (29.7)	
PLT < 85 or ≥ 300*10^9/L [n(%)]	0	237 (76.9)	549 (76.5)	0.930
	1	71 (23.1)	169 (23.5)	
ALB < 35 g/L [n(%)]	0	130 (42.2)	303 (42.2)	1.000
	1	178 (57.8)	415 (57.8)	
HbA1c > 42.07mmol /mol [n(%)]	0	40 (13.0)	107 (14.9)	0.481
	1	268 (87.0)	611 (85.1)	

Neu, Neutrophil; L, Lymphocyte; PLT, Platelet; ALB, Albumin; HbA1c, glycosylated hemoglobin

Diabetes mellitus was considered present if past medical history was diagnosed. The severe CAP was diagnosed according to the guidelines issued by the Infectious Diseases Society of America/American Thoracic Society (IDSA/ATS) and defined if (1) met one of the major criteria: acute respiratory failure requiring invasive mechanical ventilation, septic shock with the need for vasopressors; (2) met at least three minor criteria: respiratory rate ≥ 30 bpm, a ratio of partial pressure of arterial oxygen to fraction of inspired oxygen (PaO_2_/FiO_2_) ≤ 250, blood urea nitrogen (BUN) ≥ 20 mg/dL, white blood cell count < 0.4 × 10^9^/L, platelet count < 100 × 10^9^/L, body temperature < 36 ℃, multi-lobar infiltrates, confusion/disorientation, and hypotension requiring aggressive fluid resuscitation [14].

Exclusion criteria were age less than 18 years, human immunodeficiency virus infection, and active tuberculosis. Specifically, this study did not include patients with COVID-19.

### Data collection

We collected data on demographic characteristics (sex, age, and history of smoking and alcohol consumption), comorbidities (hypertension, chronic respiratory disease, cardiac dysfunction, cerebrovascular disease, chronic kidney dysfunction, rheumatic disorders, and malignant tumor), and the history of long-term glucocorticoid medication by thoroughly reviewing medical records. Glucorticosteroid administration over 20 mg/day for two weeks in the previous month was a history of long-term glucocorticoid medication. Laboratory data of routine blood tests and serum albumin levels during the first 24 h after the diagnosis of CAP were recorded.

### Sample size calculation and statistical analysis

In our previous investigation, about 11% of patients with diabetic pneumonia developed severe pneumonia, so the ratio of non-severe to severe pneumonia was 8:1. Assuming that the index of moderate diagnostic effect (AUC = 0.7) can be found, 336 non-severe and 42 severe pneumonia patients need to be included. Our training set takes twice the calculated sample size, which can meet the detection requirements.

In the study, continuous variables of laboratory tests were classified as normal or abnormal according to the clinically normal range. The data are reported as No. (%) for categorical variables, respectively. Categorical data were tested using the chi-square (χ2) test. Of the clinical baseline characteristics, univariate logistic regression analysis was used to assess the significance of each variable in training set for the prediction of severe CAP development. All variables with P < 0.05 in the univariate logistic regression analysis were incorporated into a multivariate logistic regression analysis. The nomogram for predicting severe CAP development was established based on the multivariate logistic regression analysis results and other predictors with clinical relevance. To evaluate the prediction performance of the nomogram, the area under the curve (AUC) of the receiver operating characteristic (ROC) curve and calibration with 1000 bootstrap samples were established to measure the discrimination ability to distinguish between non-severe and severe CAP patients. Calibration plots were generated to measure calibration consistency between the predicted probability and observed frequency of patients with severe CAP. The optimal cut-off values were determined by maximizing the Youden index. Decision curve analysis (DCA) based on the net benefit was depicted to show a graphical plot of net benefit against threshold probability. All statistical analyses were performed using R version 4.1.2. This report followed the Transparent Reporting of a Multivariable Prediction Model for Individual Prognosis or Diagnosis guidelines.

## Results

### Patient clinical characteristics data

**Fig. 1:**
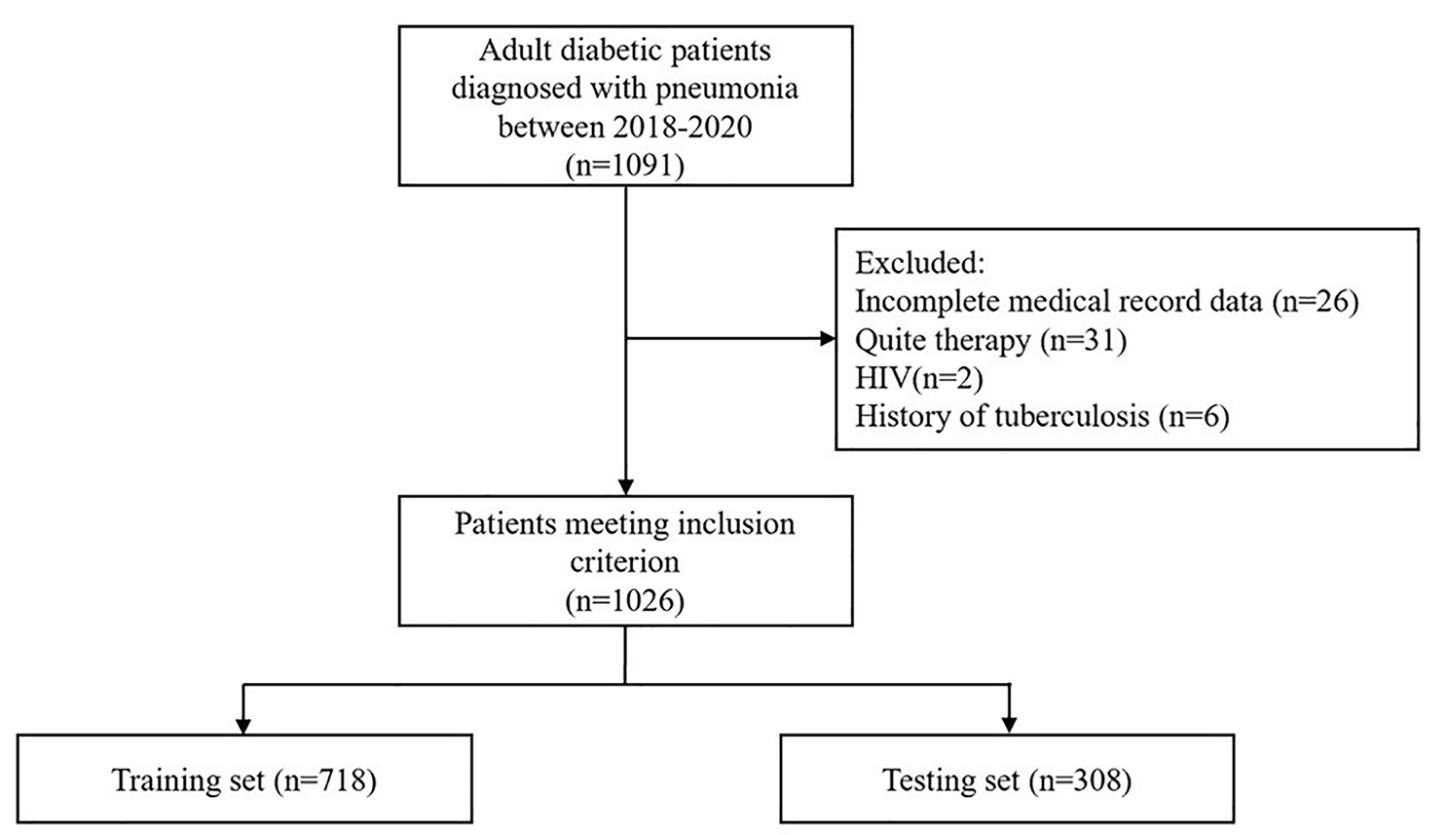
Study flow chart

A total of 1091 CAP patients with DM were included, and 1026 patients (443 women and 583 men) met the inclusion criteria (Fig. 1). The patients were divided into the training set (N = 718) and the testing set (N = 308) with a ratio of 7:3. The demographic and clinical characteristics of the training set and testing set are shown in Table 1. 79 patients in the training set and 34 in the testing set, respectively, progressed to severe CAP. There was no significant difference in the distribution of variables between the training and testing sets.

### Comparison of the indicators between DM patients with severe CAP and non-severe CAP in the training set

As shown in Table 2, the age and sex showed no significant difference between the severe and non-severe pneumonia groups. Patients who developed severe CAP had higher rates of chronic respiratory disease (26.6% vs. 15.2%), cardiac dysfunction (16.4 vs. 31.6%), chronic kidney dysfunction (5.6% vs. 21.5%) and malignant tumor (5.2% vs. 19%). Patients who received prior glucocorticoid treatment (2.2% vs. 7.6%) had higher rates of severe CAP. For clinical convenience, we transformed continuous variables of laboratory indicators into categorical variables according to the normal range. Compared to the non-severe patients, the severe CAP patients had higher rates of abnormal neutrophil count (42.3% vs. 78.5%), lymphocyte count (26.9% vs. 51.9%) and serum albumin (53.4% vs. 93.7%). Additionally, the diabetic patients who developed severe CAP showed higher levels of HbA1c than non-severe CAP patients (83.4% vs. 98.7%).

**Table 2:** Comparison of the indicators between DM patients with severe CAP and non-severe CAP in the training set

Characteristic		Non-severe CAP (n = 639)	Severe CAP (n = 79)	p
**Demographic data**				
Sex [n(%)]	Female	284 (44.4)	27 (34.2)	0.106
	Male	355 (55.6)	52 (65.8)	
Age [n(%)]	< 65	229 (35.8)	31 (39.2)	0.639
	≥ 65	410 (64.2)	48 (60.8)	
Current smoker [n(%)]	0	478 (74.9)	58 (73.4)	0.878
	1	160 (25.1)	21 (26.6)	
Alcohol abuse [n(%)]	0	593 (92.9)	74 (93.7)	0.997
	1	45 ( 7.1)	5 ( 6.3)	
Temperature < 36.3 °C or ≥ 37.2 °C [n(%)]	0	108 (16.9)	7 ( 8.9)	0.094
	1	531 (83.1)	72 (91.1)	
**Comorbid condition**				
Hypertension [n(%)]	0	296 (46.3)	27 (34.2)	0.054
	1	343 (53.7)	52 (65.8)	
Chronic respiratory disease [n(%)]	0	542 (84.8)	58 (73.4)	0.016
	1	97 (15.2)	21 (26.6)	
Cardiac dysfunction [n(%)]	0	534 (83.6)	54 (68.4)	0.002
	1	105 (16.4)	25 (31.6)	
Cerebrovascular disease [n(%)]	0	549 (85.9)	64 (81.0)	0.320
	1	90 (14.1)	15 (19.0)	
Chronic kidney dysfunction [n(%)]	0	603 (94.4)	62 (78.5)	< 0.001
	1	36 ( 5.6)	17 (21.5)	
Rheumatic disorders [n(%)]	0	627 (98.1)	76 (96.2)	0.479
	1	12 ( 1.9)	3 ( 3.8)	
Malignant tumor [n(%)]	0	606 (94.8)	64 (81.0)	< 0.001
	1	33 ( 5.2)	15 (19.0)	
Long-term glucocorticoid medication [n(%)]	0	625 (97.8)	73 (92.4)	0.017
	1	14 ( 2.2)	6 ( 7.6)	
**Laboratory data**				
Neu < 2 or ≥ 7*10^^^9/L [n(%)]	0	369 (57.7)	17 (21.5)	< 0.001
	1	270 (42.3)	62 (78.5)	
L < 0.8 or ≥ 4*10^^^9/L [n(%)]	0	467 (73.1)	38 (48.1)	< 0.001
	1	172 (26.9)	41 (51.9)	
PLT < 85 or ≥ 300*10^^^9/L [n(%)]	0	491 (76.8)	58 (73.4)	0.592
	1	148 (23.2)	21 (26.6)	
ALB < 35 g/L [n(%)]	0	298 (46.6)	5 ( 6.3)	< 0.001
	1	341 (53.4)	74 (93.7)	
HbA1c > 42.07mmol/mol [n(%)]	0	106 (16.6)	1 ( 1.3)	0.001
	1	533 (83.4)	78 (98.7)	

Neu, Neutrophil; L, Lymphocyte; PLT, Platelet; ALB, Albumin; HbA1c, glycosylated hemoglobin

### Analysis of the risk factors for progression to severe CAP in DM patients with pneumonia admitted

We performed a logistic regression analysis to determine early prediction factors of severe CAP development in DM patients with CAP admitted. With regard to comorbid conditions, chronic kidney dysfunction and malignant tumor were independently associated with severe CAP development in DM patients. Four risk factors related to laboratory examination were associated with severe CAP development in DM patients: abnormal neutrophil count, abnormal lymphocyte count, decreased serum albumin level, and increased HbA1c level (Table 3).

**Table 3:** Multivariable logistic regression analysis of risk factors for severe CAP in patients with DM in the training set

	OR(95%CI)	p
Comorbid condition		
Chronic respiratory disease	1.46 (0.77–2.78)	0.25
Cardiac dysfunction	1.23 (0.66–2.28)	0.52
Chronic kidney dysfunction	3.64 (1.71–7.76)	0.00
Malignant tumor	3.17 (1.45–6.92)	0.00
Long-term glucocorticoid medication	2.48 (0.76–8.05)	0.13
Laboratory data		
Neu < 2 or ≥ 7*10^9/L	3.26 (1.78–5.97)	0.00
L < 0.8 or ≥ 4*10^9/L	2.11 (1.23–3.64)	0.01
ALB < 35 g/L	8.09 (3.15–20.82)	0.00
HbA1c > 42.07mmol /mol	11.27 (1.51–84.25)	0.02

### Development and validation of a nomogram for severe CAP prediction in DM patients

**Fig. 2:**
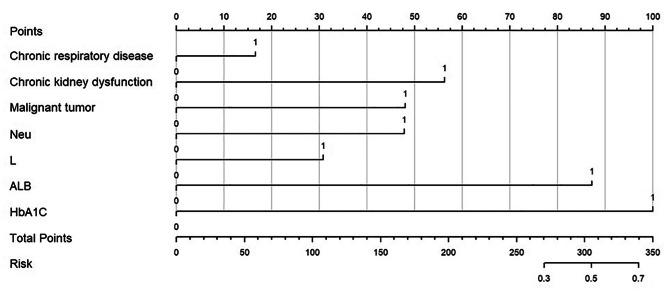
Nomogram for predicting severe CAP development in diabetic patients with CAP

**Fig. 3:**
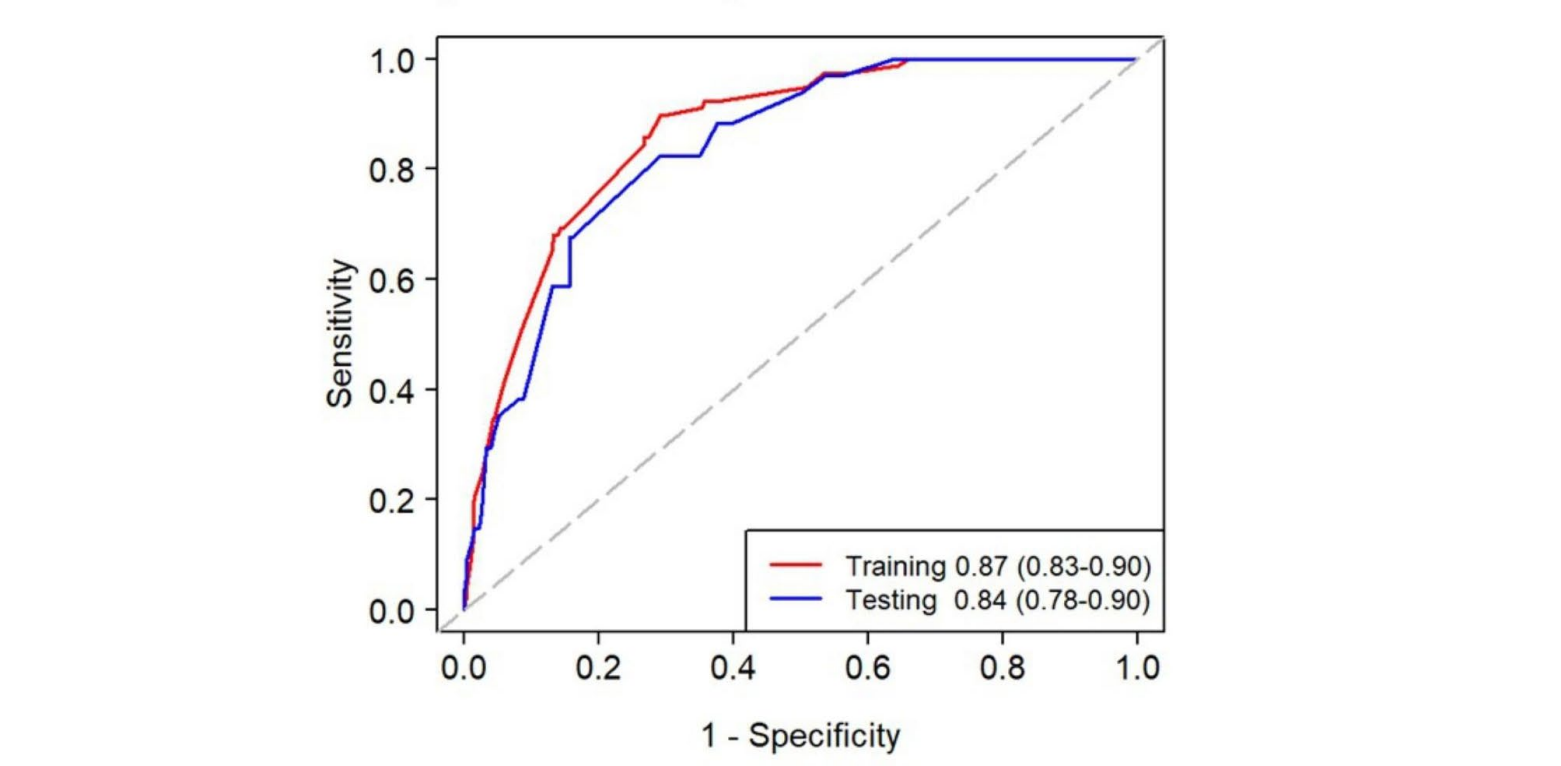
The area under the receiver operating characteristic curve (AUC) values for the prediction of severe CAP in the training set and the testing set

The nomogram was established based on the results of multivariate logistic regression analysis and other predictors with clinical relevance. The nomogram included chronic respiratory disease, chronic kidney dysfunction, malignant tumor, abnormal neutrophil count, abnormal lymphocyte count, decreased serum albumin level, and increased HbA1c level (Fig. 2). The probability of severe CAP can be estimated by calculating the total score for each patient using this nomogram. A higher score of total points indicated a greater chance of severe CAP progression in DM patients. The ROC curve was employed to assess the predictive ability of the established nomogram, and the result demonstrated that the AUC was 0.87 (95% CI 0.83–0.90) in the training set, with a sensitivity of 90% and specificity of 71% (Fig. 3; Table 4).

**Table 4:** Accuracy of the nomogram in predicting the risk of severe CAP at the optimal threshold value

Variable	Value (95%CI)	
	**Training set**	**Testing set**
Accuracy	0.90 (0.88, 0.92)	0.89 (0.85, 0.93)
AUC	0.87 (0.83, 0.90)	0.84 (0.78, 0.90)
Sensitivity	0.90 (0.81, 0.95)	0.82 (0.66, 0.92)
Specificity	0.71 (0.67, 0.74)	0.71 (0.65, 0.76)
Positive predictive value	0.63 (0.41, 0.80)	0.57 (0.20, 0.88)
Negative predictive value	0.91 (0.88, 0.93)	0.90 (0.86, 0.93)
Positive likelihood ratio	13.63 (6.17, 30.11)	10.75 (2.51, 45.99)
Negative likelihood ratio	0.82 (0.74,0.91)	0.89 (0.79, 1.01)

**Fig. 4:**
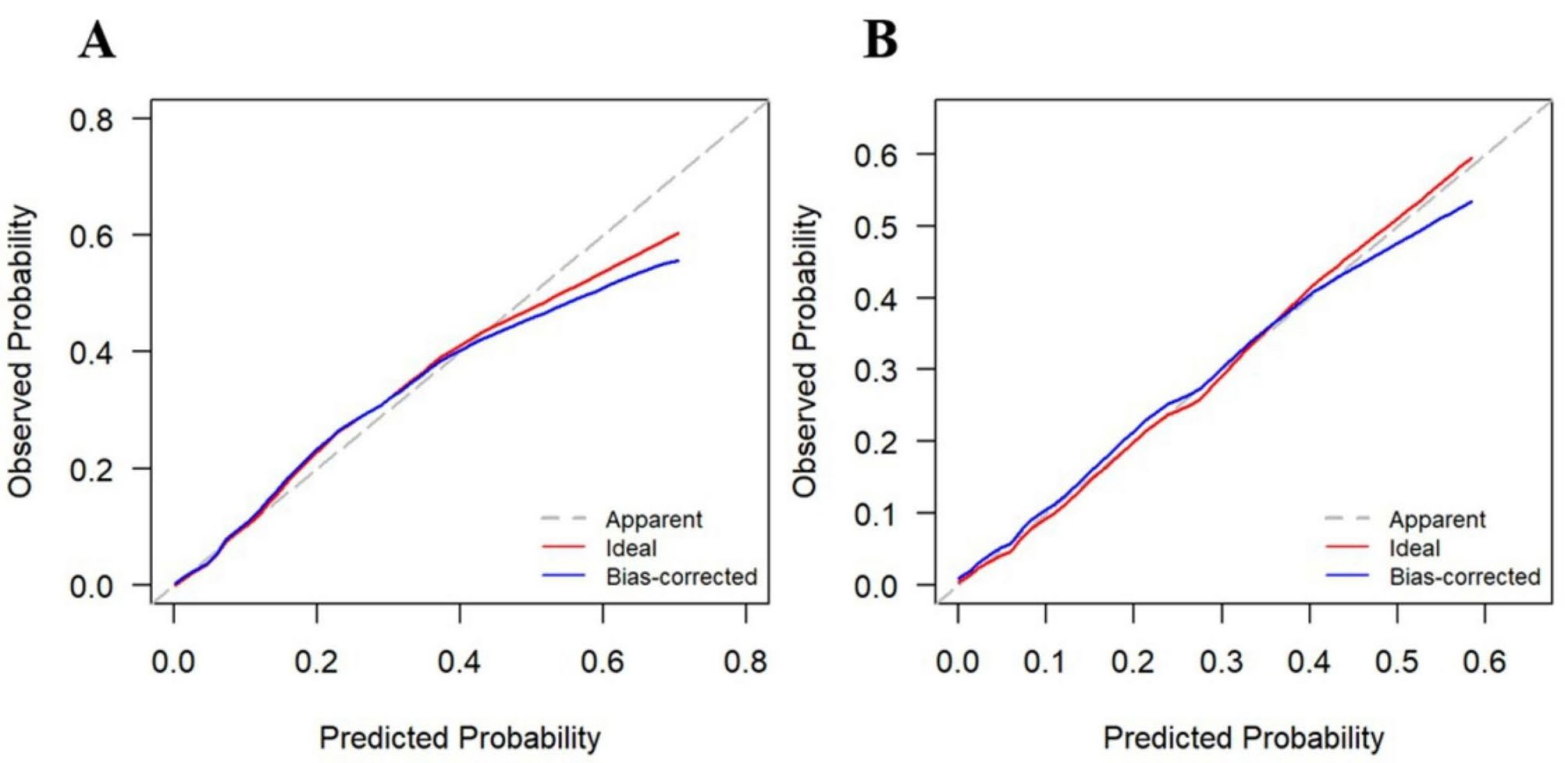
Calibration curve analysis in the training set **(A)** and testing set **(B)**

Moreover, in the testing set, ROC analysis indicated that the AUC of the nomogram was 0.84 (95% CI 0.78–0.90), with a sensitivity of 82% and specificity of 71% (Fig. 3; Table 4). Calibration curves were generated to evaluate the calibration of the prediction model. Calibration curves demonstrate acceptable model calibration with good agreement between observed frequency and predicted probability of severe CAP patients in both datasets (Fig. 4).

### Clinical application of the nomogram

**Fig. 5:**
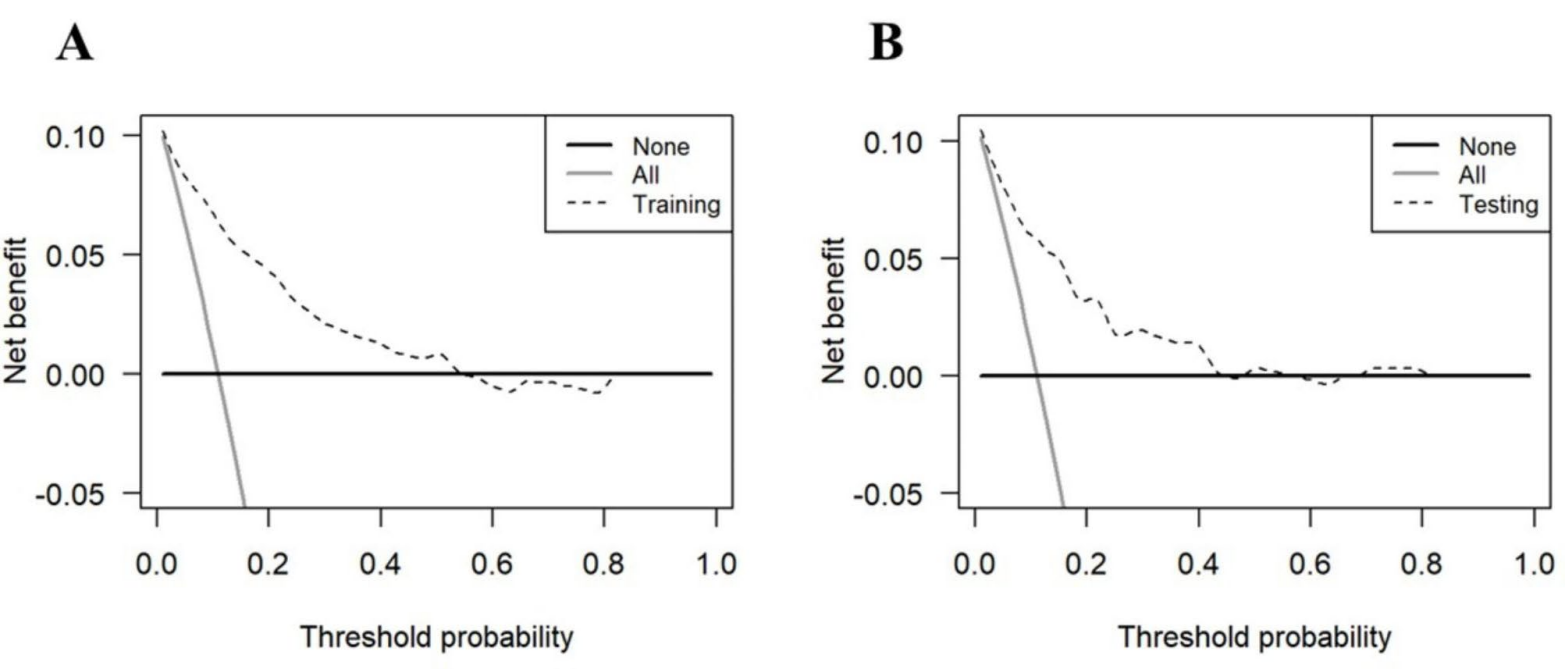
Decision curve analysis of the nomogram for predicting severe CAP in the training set **(A)** and the testing set **(B)**

We established a DCA (decision curve analysis) based on the net benefit and threshold probabilities to assess the clinical applicability of the risk prediction nomogram. The result indicated that our risk prediction nomogram was applicable with a wide range of threshold probabilities in the training and testing sets due to the net benefit (Fig. 5).

## Discussion

As known, patients with diabetes are considered to have immunocompromised status and are more prone to have chronic organ dysfunction (such as cardiovascular and kidney function). Therefore, diabetic patients have a high risk of developing severe CAP once diagnosed as CAP. Identifying these patients early and preventing them from progressing to severe CAP is essential to improve clinical outcomes. However, there is still a lack of useful tools to predict the severity risk of the specific population until now. Therefore, our study was conducted to develop a nomogram for predicting severe CAP among diabetic patients to guide treatment decisions. The nomogram incorporated routinely collected clinical data for dichotomous variables, including chronic respiratory disease, chronic kidney dysfunction, malignant tumor, abnormal neutrophil count, abnormal lymphocyte count, decreased serum albumin level, and increased HbA1c level. The nomogram showed good discrimination and calibration abilities in the training and testing sets. This nomogram would allow for easy scoring of the risk probability of developing severe CAP among DM patients in daily clinical practice. Furthermore, it would help physicians differentiate risk management for DM patients who develop CAP by weighing the net benefit of individualized clinical decision-making.

Hyperglycemia is associated with the immune system and may significantly affect the development of the severe CAP. First, hyperglycemia could reduce the mobilization of polymorphonuclear leukocytes, phagocytic activity, and chemotaxis [15]. Second, some studies found that HbA1c < 8.0% may aid to enhance CD4^+^ T cell response to foreign antigens [16]. Third, in diabetic patients, glycation of immunoglobulin was correlated with an increased level of HbA1c, which would impair the biological function of antibodies [17]. In our study, HbA1c was significantly higher in the severe CAP group than in the non-severe CAP group. HbA1c was confirmed an association with the progression of severe CAP by multivariate analysis. A study by Schuetz et al. evaluated the role of hyperglycemia in CAP patients [18]. The results suggested that in the non-critical-care setting, initial hyperglycemia was associated with a significant inflammatory response in non-diabetic patients with CAP. However, the association between hyperglycemia at admission and outcome was not found in the diabetic population. It is worthy of being noted that in the diabetic population, persistent hyperglycemia (over 96 h after admission) was associated with adverse clinical outcomes and abnormal levels of CRP.

The concept of PIRO (Predisposition, Insult, Response, and Organ dysfunction) was introduced in 2003 to help develop a stratified system for sepsis [19]. In this study, we hypothesize that the management of DM patients who are developing CAP follows the development of a staging system inspired by the PIRO concept, which can improve individualized clinical decision-making based on predisposing factors and premorbid conditions and extend to the resultant organ dysfunction. Moreover, patients with diabetes are commonly comorbid with chronic complications (such as cardiovascular disease, renal failure, and pulmonary microangiopathy), which may also influence the progression of CAP and prognosis [6]. In our study, a higher proportion of chronic respiratory diseases, cardiac dysfunction, chronic kidney dysfunction, and malignant tumor were found in severe CAP patients. It was also reported that DM complications might be an additional risk factor for pneumonia in this population [20]. Patients with chronic comorbidities such as chronic respiratory disease and cardiac dysfunction are at increased risk of death from CAP over the short and long term [21].

Usually, CAP occurs when the immune system is compromised and encounters a microbial infection. Several variables associated with the immune system have been demonstrated as risk factors of CAP or biomarkers of CAP severity. Lymphocytes are crucial for immunity. It has been shown that diabetic patients have reduced germinal centers and decreased pathogen-specific Th17 cells, which causes downstream lymphocyte dysfunction [15]. In hospitalized CAP patients, lymphopenia was a risk factor for mortality, and lymphocyte count could improve the prediction accuracy of the CURB-65 score for 30-day mortality [22]. Our study confirmed that abnormal lymphocyte count was the risk factor for severe CAP development in DM patients. In a study by Bermejo-Martin et al., patients with lymphocyte < 724/mm^3^ had increased 30-day mortality by 1.93-fold in hospitalized CAP patients, irrespective of the CURB-65 score, critical illness, and appropriate antibiotic treatment [22].

The neutrophil count has presently been considered a readily available indicator of infection in the acute care setting of the emergency department [23]. In our study, abnormal neutrophils count can help predict severe CAP progression in DM patients with CAP admission. In immunocompromised patients with severe pneumonia, higher neutrophils count was also independently associated with hospital mortality [24]. Several studies have shown impaired neutrophil function, an important mechanism contributing to the high incidence of infection in diabetic patients [15, 25]. It was reported that the migration, phagocytosis, and microbial killing of neutrophils in DM patients are impaired [15]. The abnormal neutrophil count has been reported in many studies to be associated with severity and poor prognosis in patients with pneumonia.

Several studies have confirmed that serum albumin can be used as a prognostic marker for the critical ill disease. In the case of diabetes mellitus, albumin synthesis relies on adequate insulin reserves [26]. Diabetes is associated with an increased risk of malnutrition [27]. DM patients with malnutrition features such as low serum albumin have more comorbidities, including dementia [28]. In our study, decreased serum albumin level was independently associated with severe CAP development in DM patients. Poor nutritional status may indicate a high risk of pneumonia in older adults [29]. Moreover, patients with preoperative hypoalbuminemia have been reported to be at higher risk for surgical site infection, pneumonia, and prolonged hospital stay after total arthroplasty [30].

It is vital to develop a prediction tool for early identification of progression to severe pneumonia. Until now, several predictive tools have been characterized in managing pneumonia. CURB65 and PSI scores are currently the most important scoring systems used to assess CAP in the clinic [31]. The CURB65 and PSI scores are the most widely used and are recommended by multiple CAP guidelines for assessing CAP. CURB65 has few items and is easy to remember and operate. PSI requires too many statistical variables and is more complex and difficult to complete quickly, which limits its widespread clinical use to a certain extent. However, few studies have explored the efficacy of CURB65 and PSI scores in predicting the severity of pneumonia in diabetic patients. Several studies showed that the performance of CURB-65 and PSI scores in predicting in-hospital mortality was reduced in diabetic patients compared with non-diabetic patients [12, 32]. Ma et al. found that the AUCs of CURB65 for predicting in-hospital mortality were 0.757 in patients without T2DM and 0.677 in patients with T2DM (P < 0.001). In addition, the AUCs of PSI for predicting in-hospital mortality were 0.798 in patients without T2DM and 0.716 in patients with T2DM (P < 0.05) [12]. This means that CURB65 and PSI scores do not perform well in diabetic patients with pneumonia. There is a clinical need for a predictive score for pneumonia severity in patients with diabetes mellitus. Our study showed the AUC of our nomogram is 0.84, indicating that our nomogram performs well in predicting the pneumonia severity in diabetic patients. The study by Cheng et al. reported that the AUC of PSI in predicting in-hospital mortality in diabetic patients with CAP were 0.854, which was slightly superior to that of our nomogram [32]. The main difference between this study and ours was the difference in the predicted primary outcome. In our study, we mainly analyzed the efficacy of our nomogram in predicting severe pneumonia, while Cheng’s study explored the efficacy of PSI in predicting in-hospital mortality. The PSI contains 18 items and stratifies patients into five categories, which can effectively predict the risk of death of patients. The main implication of our model is to predict the occurrence of severe CAP in patients with diabetic pneumonia. In addition, A clear advantage of our scoring system is its simplicity and convenience. It is based on readily available dichotomous variables, all of which have known effects on CAP progression. To our knowledge, most scoring systems that predict the severity risk of CAP do not consider the pathological features of specific diseases or comorbidities, leading to heterogeneity in patients and inaccurate outcomes results. Our scoring system incorporates HbA1c and comorbid conditions to identify the risk of severe CAP development early in DM patients.

Although several interesting findings were found, our study remains to have several limitations. First, the lack of comparison between our nomogram and existing scores is a major flaw in our study. Second, selection bias may exist due to the study’s retrospective nature, and the risk for confounding factors may be indicated. Third, for comorbidities, we cannot be clear that this was a complication caused by diabetes. Therefore, comorbidities do not reflect the severity of diabetes and the stage of the course. Fourth, it was well known that clinical signs and symptoms of patients play an essential role in developing severe CAP. However, the present nomogram model was not included clinical signs and symptoms such as chest tightness and dyspnea, given that clinical symptoms were subjective.

## Conclusion

In conclusion, based on the clinical data with dichotomous variables within 24 h of onset, we have made a nomogram to predict severe CAP development in DM patients with CAP. The nomogram has good prediction accuracy and discrimination ability and can be applied to estimate early the probabilities of progression of severe CAP in DM patients. Nonetheless, further studies with larger sample sizes or prospective cohort studies are needed to evaluate and validate the current nomogram.

## Data Availability

All generated or analyzed data in this study are included in the published article.
